# Vitamin C Depletion and All-Cause Mortality in Renal Transplant Recipients

**DOI:** 10.3390/nu9060568

**Published:** 2017-06-02

**Authors:** Camilo G. Sotomayor, Michele F. Eisenga, Antonio W. Gomes Neto, Akin Ozyilmaz, Rijk O. B. Gans, Wilhelmina H. A. de Jong, Dorien M. Zelle, Stefan P. Berger, Carlo A. J. M. Gaillard, Gerjan J. Navis, Stephan J. L. Bakker

**Affiliations:** 1 Department of Internal Medicine, University Medical Center Groningen, University of Groningen, Hanzeplein 1, Groningen 9700 RB, The Netherlands; m.f.eisenga@umcg.nl (M.F.E.); a.w.gomes.neto@umcg.nl (A.W.G.N.); a.ozyilmaz@umcg.nl (A.O.); r.o.b.gans@umcg.nl (R.O.B.G.); d.m.zelle@umcg.nl (D.M.Z.); s.p.berger@umcg.nl (S.P.B.); c.a.j.m.gaillard@umcg.nl (C.A.J.M.G.); g.j.navis@umcg.nl (G.J.N.); s.j.l.bakker@umcg.nl (S.J.L.B.); 2 Department of Laboratory Medicine, University Medical Center Groningen, University of Groningen, Hanzeplein 1, Groningen 9700 RB, The Netherlands; w.h.a.de.jong@umcg.nl

**Keywords:** renal transplant, vitamin C, mortality, inflammation, hs-CRP

## Abstract

Vitamin C may reduce inflammation and is inversely associated with mortality in the general population. We investigated the association of plasma vitamin C with all-cause mortality in renal transplant recipients (RTR); and whether this association would be mediated by inflammatory biomarkers. Vitamin C, high sensitive C-reactive protein (hs-CRP), soluble intercellular cell adhesion molecule 1 (sICAM-1), and soluble vascular cell adhesion molecule 1 (sVCAM-1) were measured in a cohort of 598 RTR. Cox regression analyses were used to analyze the association between vitamin C depletion (≤28 µmol/L; 22% of RTR) and mortality. Mediation analyses were performed according to Preacher and Hayes’s procedure. At a median follow-up of 7.0 (6.2–7.5) years, 131 (21%) patients died. Vitamin C depletion was univariately associated with almost two-fold higher risk of mortality (Hazard ratio (HR) 1.95; 95% confidence interval (95%CI) 1.35–2.81, *p* < 0.001). This association remained independent of potential confounders (HR 1.74; 95%CI 1.18–2.57, *p* = 0.005). Hs-CRP, sICAM-1, sVCAM-1 and a composite score of inflammatory biomarkers mediated 16%, 17%, 15%, and 32% of the association, respectively. Vitamin C depletion is frequent and independently associated with almost two-fold higher risk of mortality in RTR. It may be hypothesized that the beneficial effect of vitamin C at least partly occurs through decreasing inflammation.

## 1. Introduction

Renal transplantation is currently considered the “gold standard” treatment for end-stage renal disease (ESRD) patients, since it offers superior survival, quality of life and cost-effectiveness compared to chronic dialysis treatment [1,2,3,4,5,6,7,8]. Nevertheless, survival of renal transplant recipients (RTR) is significantly lower than of age-matched controls in the general population [9]. 

It is worth noting that after renal transplantation a long-term ongoing inflammatory status persists [10,11,12]. It was recently reported that higher inflammatory status is associated with an increased risk of mortality in RTR [13]. In keeping with this finding, high sensitive C-reactive protein (hs-CRP), an established marker of inflammation, has been associated with increased risk of mortality in RTR [14,15]. It has been reported that vitamin C (ascorbic acid) is negatively correlated with C-reactive protein [16]. Both oral and high-dose intravenous vitamin C therapy reduced CRP levels and other pro-inflammatory cytokines [17,18,19]. Furthermore, vitamin C has been shown to be inversely associated with risk of all-cause mortality in the general population [20,21,22,23,24]. However, to date the role and long-term effects of vitamin C status on inflammatory biomarkers and adverse outcomes such as all-cause mortality in stable RTR remains unexplored, yet the results are of significant interest.

In this study, we aimed to investigate prospectively whether plasma vitamin C concentration and, specifically, its depletion (≤28 µmol/L) [25,26,27,28,29,30,31] is associated with risk of all-cause mortality in RTR. In addition, we aimed to evaluate whether a putative association between vitamin C concentration and risk of all-cause mortality in RTR would be mediated by inflammatory parameters such as hs-CRP, soluble intercellular cell adhesion molecule 1 (sICAM-1) and soluble vascular cell adhesion molecule 1 (sVCAM-1).

## 2. Materials and Methods 

### 2.1. Study Design

In this prospective cohort study, all adult RTR who survived with a functioning allograft beyond the first year after transplantation, and without known or apparent systemic illnesses (i.e., malignancies, opportunistic infections) were invited to participate during their next visit to the outpatient clinic. From a total of 847 eligible RTR, 606 (72%) patients signed informed consent. The group that did not sign informed consent was comparable with the group that signed informed consent with respect to age, sex, body mass index (BMI), serum creatinine, creatinine clearance, and proteinuria. Baseline data was collected between August 2001 and July 2003 at a median 5.9 (interquartile range (IQR): 2.6–11.4) years after renal transplantation. For the statistical analyses we excluded patients missing plasma vitamin C measurements (*n* = 8), resulting in 598 RTR eligible for analyses. Use of vitamin C supplements or multivitamin supplements containing vitamin C were documented in all RTR. The Institutional Review Board approved the study protocol (METc 2001/039). The clinical and research activities being reported are consistent with the Principles of the Declaration of Istanbul as outlined in the ‘Declaration of Istanbul on Organ Trafficking and Transplant Tourism’.

The primary endpoint of this study was RTR mortality of all cause in nature. The continuous surveillance system of the outpatient program ensures up-to-date information on patient status. We contacted general practitioners or referring nephrologists in case the status of a patient was unknown. There was no loss due to follow-up. 

### 2.2. Renal Transplant Characteristics

Relevant transplant characteristics including both donor and recipient age and gender, as well as transplant information were extracted from the Groningen Renal Transplant Database, which contains information about all renal transplantations that have been performed at the University Medical Centre Groningen since 1986. Smoking status was obtained using a self-report questionnaire. Smoking behavior was classified as never, former or current smoker. Cardiovascular disease history was considered positive if participants had a myocardial infarction, transient ischemic attack or cerebrovascular accident. Data on cumulative dose of steroids, incidence of acute rejection episodes and use of mechanistic target of rapamycin (m-TOR) inhibitors were retrieved from individual patient files. Cumulative dose of prednisolone was calculated as the sum of maintenance dose of prednisolone until inclusion and the dose of prednisolone or methylprednisolone required for treatment of acute rejection (a conversion factor of 1.25 was used to convert methylprednisolone dose to dose of prednisolone).

### 2.3. Measurements

Body mass index was calculated as weight in kilograms, divided by height in meters squared. Waist circumference was measured on bare skin midway between the iliac crest and the 10th rib. Blood pressure was measured as the average of three automated (Omron M4, Omron Europe B.V., Hoofddorp, The Netherlands) measurements with 1-min intervals after a 6-min rest in supine position. 

Blood was drawn in the morning after an 8 to 12 h overnight fasting period, which included no medication intake. In order to measure plasma vitamin C concentration, blood was directly after phlebotomy transferred to the laboratory on ice, deproteinized and stored in the dark at –20 °C until analysis. For quantitative measurement ascorbic acid is enzymatically transformed to dehydroascorbic acid, which in turn is derivatized to 3-(1,2-dihydroxyethyl)furo-[3,4-b]quinoxaline-1-one. Then, reversed phase liquid chromatography with fluorescence detection is applied (excitation 355 nm, emission 425 nm). Serum high sensitive C-reactive protein was assessed as described before [32]. Plasma sICAM-1 and sVCAM-1 concentrations were measured by enzyme-linked immunosorbent assay kits (Diaclone Research, Besançon, France). Serum creatinine concentrations were determined using the Jaffé method (MEGA AU510, Merck Diagnostica, Darmstadt, Germany). Total cholesterol was determined using the cholesterol oxidase-phenol aminophenazone method (MEGA AU510, Merck Diagnostica, Darmstadt, Germany), and serum triglycerides were determined with the glycerol-3-phosphate oxidase-phenol aminophenazone method (MEGA AU510, Merck Diagnostica, Darmstadt, Germany). High density lipoprotein (HDL)-cholesterol was determined with the cholesterol oxidase-phenol aminophenazone method on a Technikon RA-1000 (Bayer Diagnostics, Mijdrecht, The Netherlands), and low density lipoprotein (LDL)-cholesterol was calculated using the Friedewald formula [33]. Plasma glucose was determined by the glucose-oxidase method (YSI 2300 Stat plus, Yellow Springs, OH, USA). Glycated hemoglobin (HbA1c) was determined by high performance liquid chromatography (VARIANTTM HbA1c Program with Bio-Rad CARIANT Hb Testing System, Bio-Rad, Hercules, CA, USA). 

According to a strict protocol all RTR were asked to collect a 24-hour urine sample during the day before their visit to the outpatient clinic. Urine was collected under oil and chlorohexidine was added as an antiseptic agent. Proteinuria was defined as urinary protein excretion >0.5 g/24 h. Renal function was assessed by estimated Glomerular Filtration Rate (eGFR) applying the Chronic Kidney Disease Epidemiology Collaboration equation [34]. 

### 2.4. Statistical Analysis

Data were analyzed using IBM SPSS software version 23.0 (SPSS Inc., Chicago, IL, USA), STATA 12.0 (StataCorp LP, College Station, TX, USA) and R version 3.2.3. In all analyses, a 2-sided *p* < 0.05 was considered significant. Hazard ratio (HR) are reported with 95% confidence interval (CI). Continuous variables were summarized using mean (standard deviation (SD)) for normally distributed data, whereas skewed distributed variables are given as median (IQR); percentages were used to summarize categorical variables. Linear regression analyses were performed to evaluate the association of plasma vitamin C concentration with recipient-related and transplantation-related characteristics. Natural log transformation was used for analyses of variables with a skewed distribution. 

A log-rank test was run to determine if there were differences in the survival distribution between plasma vitamin C status (depleted and non-depleted; ≤ or >28 µmol/L, respectively) of RTR. To analyze whether plasma vitamin C concentration is independently associated with mortality, we performed Cox-proportional hazards regression analyses. For these analyses plasma vitamin C concentration was used as categorical variable according to depleted or not depleted concentration [25,26,28,29,30]; and as continuous variable (2 base of log-transformed values to achieve a normal distribution), in order to obtain the best fitting model. First, we performed univariate Cox regression analyses. Hereafter, we adjusted for age and sex (Model 2); for eGFR, proteinuria, primary renal diseases and time since transplantation (Model 3). To avoid inclusion of too many variables for the number of events, further models were performed with additive adjustments to model 3. We performed additional adjustments for smoking status and alcohol use (Model 4); for diabetes mellitus (Model 5); and for systolic blood pressure, BMI, serum HDL cholesterol and triglycerides concentration (Model 6); and for use of calcineurin inhibitors, use of antimetabolites, use of m-TOR inhibitors, use of induction therapy, and cumulative dose of prednisolone (model 7). 

As secondary analyses, we also performed classic mediation analyses according to Preacher and Hayes [35,36], which are based on logistic regression; to establish whether hs-CRP sICAM-1 and sVCAM-1 concentrations, separately and combined (sum of individual *Z* scores of hs-CRP + sICAM-1 + sVCAM-1), mediated the association between plasma vitamin C concentration and all-cause mortality. These analyses allow for testing significance and magnitude of mediation. 

## 3. Results

### 3.1. Baseline Characteristics

A total of 598 stable RTR were included (mean age 51 ± 12 years, 54% male, 96% caucasian) at 5.9 (2.6–11.4) years after transplantation. Among them 133 (22%) RTR were vitamin C depleted. None of the patients used vitamin C supplements or multivitamin supplements containing vitamin C. Median (IQR) plasma vitamin C, hs-CRP, sICAM-1 and sVCAM-1 concentration were 44 (31–55) µmol/L, 2.0 (0.7–4.8) mg/L, 602 (514–720) ng/L, and 965 (772–1196) ng/L, respectively. Mean eGFR was 47 ± 15 mL/min/1.73 m^2^, 166 (28%) participants had proteinuria. Additional baseline characteristics are shown in Table 1.

### 3.2. Association of Plasma Vitamin C Concentration with Clinical Variables

Age- and sex-adjusted plasma vitamin C concentration was associated with hs-CRP (std. β = −0.19; *p* < 0.001), sICAM-1 (std. β = −0.17; *p* < 0.001) and sVCAM-1 (std. β = −0.16; *p* < 0.001) concentrations. Moreover, alkaline phosphatase (std. β = −0.21; *p* < 0.001) and gamma glutamate (std. β = −0.10; *p* = 0.02) were associated with plasma vitamin C concentration. Furthermore, vitamin C was significantly associated with HbA1c (std. β = −0.12; *p* = 0.002), diabetes (std. β = −0.11; *p* = 0.008), and insulin concentration (std. β = −0.08; *p* = 0.04). Likewise, eGFR (std. β = 0.11; *p* = 0.009), systolic blood pressure (std. β = −0.11; *p* = 0.004) and diastolic blood pressure (std. β = −0.11; *p* = 0.01) were associated to vitamin C concentration. Dialysis vintage (std. β = −0.14; *p* = 0.001) and immunosuppressive therapy including use of calcineurin inhibitors (std. β = −0.09; *p* = 0.02), use of m-TOR inhibitors (std. β = −0.10; *p* = 0.02), induction therapy (std. β = −0.20; *p* < 0.001), acute rejection treatment (std. β = −0.13; *p* = 0.03), and cumulative dose of prednisolone (std. β = −0.21; *p* ≤ 0.001), were associated to plasma vitamin C concentration (Table 1). 

### 3.3. Prospective Analyses

**Figure 1:**
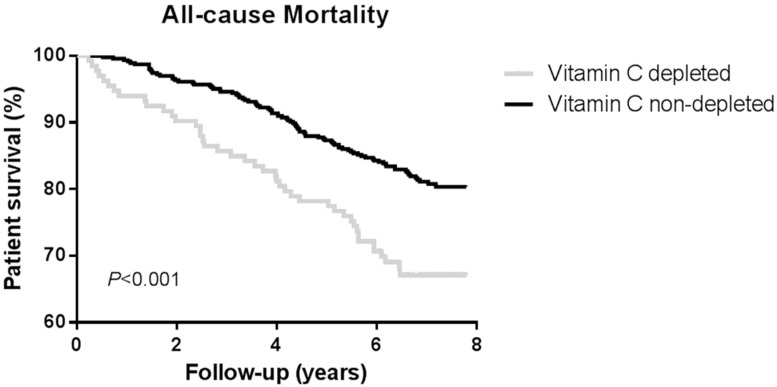
Kaplan-Meier curve for all-cause mortality according to plasma vitamin C status (depleted versus non-depleted) among renal transplant recipients. Vitamin C depleted: ≤28 µmol/L; Vitamin C non-depleted: >28 µmol/L.

During a median follow-up of 7.0 (6.2–7.5) years, 131 (21%) patients died. 32% of plasma vitamin C depleted patients died, whereas among non-depleted patients 18% died. The survival distributions between depleted and non-depleted RTR were significantly different (log-rank test *p* < 0.001). A Kaplan-Meier curve for all-cause mortality according to plasma vitamin C status is shown in Figure 1. 

Results of univariate and multivariate Cox-proportional hazard regression analyses are shown in Table 2. Prospective analyses of the association between vitamin C concentration with all-cause mortality showed that plasma vitamin C depleted RTR had an almost double risk of mortality (HR 1.95; 95% CI 1.35–2.81, *p* < 0.001). This association was independent of further adjustment for potential confounders, with e.g., an HR of 1.88; 95% CI 1.28–2.76, *p* = 0.001 after adjustment for age, sex, eGFR, proteinuria, primary renal disease, time since transplantation and dialysis vintage. Further adjustment for other potential confounders (i.e., smoking and alcohol status, diabetes mellitus, systolic blood pressure, BMI, HDL cholesterol and triglycerides concentration, use of calcineurin inhibitors, use of antimetabolites, use of m-TOR inhibitors, use of induction therapy, and cumulative dose of prednisolone) did not materially alter the association.

**Figure 2:**
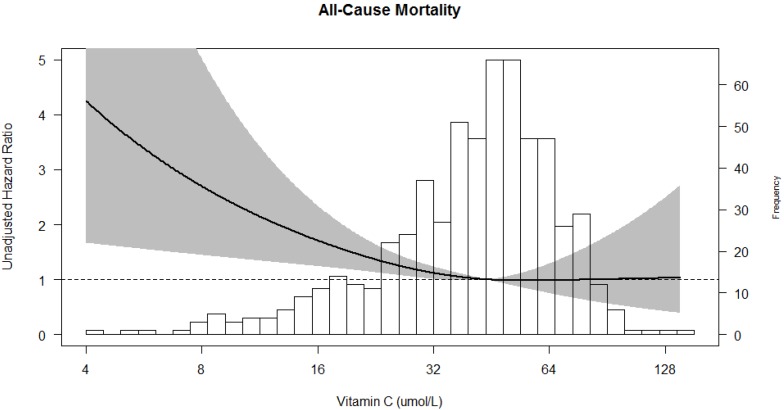
Association of plasma vitamin C with risk of all-cause mortality. The line in the graph represents the hazard ratio. The grey area represents the 95% confidence interval of the hazard ratio.

Vitamin C as a continuous variable was univariately associated with all-cause mortality (HR 0.71; 95% CI 0.59–0.87, *p* = 0.001), with the point estimate of the HR below 1.00 indicating that risk decreases with increasing vitamin C concentrations. In multivariable analysis, after adjustment for potential confounders the association remained, with a HR of 0.76; 95% CI 0.62–0.94, *p* = 0.011 (Table 2; Figure 2).

### 3.4. Mediation Analyses

**Figure A1:**
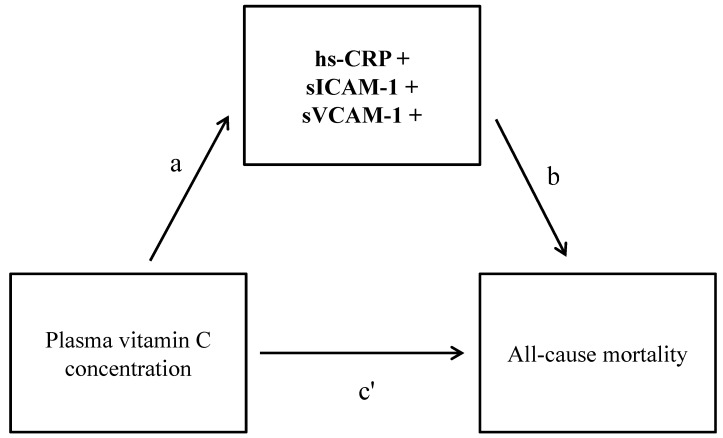
Mediation analysis of combined score between hs-CRP, sICAM-1 and sVCAM-1 on the association of plasma vitamin C concentration with all-cause mortality. *a*, *b* and *c* are the standardized regression coefficients between variables. The indirect effect (through a potential mediator) is calculated as *a* × *b*. Total effect (*c*) is *a* × *b* + *c*’. Magnitude of mediation is calculated as indirect effect divided by total effect.

In mediation analyses according to the procedures of Preacher and Hayes [35,36], hs-CRP, sICAM-1 and sVCAM-1 concentration were significant mediators (*p* value for indirect effect <0.05) in the association of vitamin C concentration with mortality. The magnitude of the mediating effects of hs-CRP, sICAM-1 and sVCAM-1 accounted 16, 17 and 15%, respectively. Furthermore, combined inflammatory biomarkers mediated 32% on the association of plasma vitamin C concentration with risk of all-cause mortality in RTR (Table 3; Figure A1).

## 4. Discussion

This study showed, first, that vitamin C depletion was common in a stable outpatient population of RTR, and that plasma vitamin C concentration was independently and inversely associated with risk of all-cause mortality in RTR. Particularly, plasma vitamin C depletion was detrimental, as depicted by an almost two fold higher risk of mortality within patients that had plasma vitamin C concentration equal or lower than 28 µmol/L [25,26,27,28,29,30,31]. Importantly, adjustment for several potential confounders did not alter the association. Of note, the association between vitamin C and mortality has been previously reported in the general population [20,21,22,23,24]; however, to our knowledge, this is the first study that examines the association of plasma vitamin C concentration with all-cause mortality in RTR and, specifically, the effect of plasma vitamin C depletion on patient survival after renal transplantation. 

Further, we found that combined inflammatory biomarkers mediated the robust proportion of about one third of the association of plasma vitamin C concentration with all-cause mortality. Notwithstanding that the underlying mechanisms leading to significantly lower survival of RTR compared to age-matched controls in the general population [9] are not completely understood, it is noteworthy that a long-term ongoing inflammatory status remains after renal transplantation [10,11,12]. Indeed, Abedini et al. [15], reported that in a cohort of 2102 RTR, over a follow-up period of 5–6 years, hs-CRP was independently associated with all-cause mortality in RTR. Likewise, Winkelmayer et al. [14] found that, at a median follow-up of 7.8 years after renal transplantation in a cohort of 438 RTR, CRP levels of more than 5 mg/L were associated with an 83% greater mortality risk compared with lower levels of this inflammatory marker. These observations are in agreement with our findings and support the influence of low-grade ongoing inflammation on patient survival after renal transplantation. On the basis of these findings and currently available literature [10,11,12,13,14,15] one might propose that inflammation plays a major role in the underlying mechanisms leading to decreased survival after renal transplantation. Finally, taking into account that we found that vitamin C concentration was inversely associated with inflammatory biomarkers, which is in agreement with previous reports [16,17,18,19], we hypothesize that the beneficial effect of adequate vitamin C status on survival of RTR is at least partly mediated by diminishing inflammatory status.

On the basis of current findings it is expected that reduction of inflammation through vitamin C supplementation, could be an approach to encourage protection against tissue injury and improve current survival rates of RTR. A recent randomized controlled trial evaluated the effect of oral vitamin C supplementation (200 mg/day during 3 months) on inflammatory status among 100 maintenance hemodialysis patients [37]. Compared with patients that did not receive supplementation, a significant decrease of hs-CRP levels was found among the vitamin C supplemented group. Moreover, the hs-CRP levels returned to their original state after the supplementation was withdraw. In turn, Atallah et al. reported the effect of intravenous vitamin C supplementation (300 mg each dialysis session) on inflammatory parameters in hemodialysis patients. This study showed that CRP levels between baseline and 6 months were significantly decreased in the supplemented, but not in the control group [38]. Nevertheless, to our knowledge no randomized controlled trial has been reported evaluating the effect of vitamin C supplementation strategies on inflammatory biomarkers or prospective outcomes in RTR.

The strength of this study lays in its prospective design; and that it comprises a large cohort of stable RTR which were closely monitored by regular check-up in the outpatient clinic, which gives complete information on patient status. A limitation is that we did not have repeated measurements of vitamin C levels. However, it should be realized that if intra-individual variability of vitamin C is taken into account, the predictive properties become stronger. The higher the intra-individual day-to-day variation of vitamin C would be, the greater one would expect the benefit of repeated measurement for prediction of outcomes [39,40]. Moreover, as with any observational study, reversed causation or unmeasured confounding may occur, despite the substantial number of potentially confounding factors for which we adjusted. As we have no data on nutrition, we cannot exclude the possibility that the association exists as a consequence of vitamin C being a marker of poor nutrition. Finally, since this is a single center study; the predictive value of vitamin C on mortality in RTR requires to be confirmed within a multicenter study. 

## 5. Conclusions

In conclusion, plasma vitamin C depletion is common in stable RTR, and is independently and inversely associated with all-cause mortality after renal transplantation. Since hs-CRP, sICAM-1 and sVCAM-1 were found to be important mediators in the association between vitamin C and all-cause mortality, we hypothesize that the beneficial effect of vitamin C would occur through decreasing inflammatory status. On the basis of the current findings, further research is needed to evaluate whether vitamin C supplementation could be a therapeutic strategy in order to increase survival after renal transplantation. The present study should encourage the design of multicenter, randomized, double-blind, placebo-controlled trial, aimed to test the efficacy of this novel therapeutic strategy.

## Figures and Tables

**Table 1:** Baseline characteristics of RTR and its association with plasma vitamin C, adjusted for age and sex.

Variables			All Patients	Vitamin C (Ln), µmol/L	
				Std. β	*p* Value
No. of patients			598	-	-
Vitamin C, µmol/L			44 (31–55)	-	-
Demographics					
	Age, years		51 ± 12	−0.05 *	0.23 *
	Sex (male), *n* (%)		328 (54)	−0.18 *	<0.001 *
	Ethnicity (caucasian), *n* (%)		577 (96)	−0.02	0.60
Body Composition					
	Body surface area, m^2^		1.87 ± 0.19	−0.04	0.22
	Body mass index, kg/m^2^		26.0 ± 4.3	−0.08	0.06
Primary Renal Diseases				−0.02	0.61
	Primary glomerulonephritis, *n* (%)		169 (28)	-	-
	Glomerulonephritis due to vascular or autoimmune disease, *n* (%)		36 (6)	-	-
	Tubulointerstitial nephritis and pyelonephritis, *n* (%)		92 (15)	-	-
	Polycystic kidney disease, *n* (%)		106 (18)	-	-
	Dysplasia and hypoplasia, *n* (%)		21 (4)	-	-
	Renovascular disease, *n* (%)		32 (5)	-	-
	Diabetic nephropathy, *n* (%)		22 (4)	-	-
	Hereditary diseases and other, *n* (%)		117 (20)	-	-
Tobacco Use				−0.08	0.06
	Never smoker, *n* (%)		214 (35)	-	-
	Ex-smoker, *n* (%)		251 (42)	-	-
	Current smoker, *n* (%)		131 (21)	-	-
Blood Pressure					
	Systolic blood pressure, mmHg		153 ± 22	−0.11	0.004
	Diastolic blood pressure, mmHg		89 ± 9	−0.11	0.01
	Use of ACE-inhibitor or aII-antagonist, *n* (%)		201 (33)	0.07	0.11
	Use of beta-blocker, *n* (%)		368 (61)	−0.07	0.11
Prior History of CV Disease					
	History of MI, *n* (%)		48 (8)	−0.01	0.75
	History of TIA/CVA, *n* (%)		32 (5)	−0.04	0.36
Transplantation					
	Time since transplantation, years		5.9 (2.6–11.4)	0.20	<0.001
	Dialysis vintage, months			−0.14	0.001
		141 (24)		-	-
		1–5 years	363 (61)	-	-
		>5 years	94 (16)	-	-
	Deceased donor, *n* (%)		515 (86)	0.02	0.61
Immunosuppressive Therapy					
	Prednisolone, mg/day		10.0 (7.5–10.0)	−0.11	0.008
	Use of calcineurin inhibitors			−0.09	0.02
		Cyclosporine, *n* (%)	386 (65)	-	-
		Tacrolimus, *n* (%)	84 (14)	-	-
		None, *n* (%)	128 (21)	-	-
	Use of antimetabolites			−0.06	0.19
		Azathioprine, *n* (%)	194 (32)	-	-
		Mycophenolic acid, *n* (%)	247 (41)	-	-
		None, (%)	157 (26)	-	-
	Use of m-TOR inhibitors, *n* (%)		10 (2)	−0.10	0.02
	Induction therapy			−0.20	<0.001
		Anti-thymocyte globulin, *n* (%)	70 (12)	-	-
		Muromonab-CD3 , *n* (%)	26 (4)	-	-
		Anti-CD25 monoclonal antibodies, *n* (%)	10 (2)	-	-
		None, *n* (%)	492 (82)	-	-
	Acute rejection treatment			−0.13	0.03
		High doses of steroids, *n* (%)	186 (31)	-	-
		Other rejection therapy, *n* (%)	82 (14)	-	-
	Cumulative dose of prednisolone, grams		21.3 (11.3–37.9)	0.21	<0.001
Ischemia Times					
	Cold ischemia time, hours		22 (15–27)	0.01	0.75
	Total warm ischemia, minutes		35 (30–45)	0.02	0.72
Renal Allograft Function					
	eGFR, mL/min/1.73 m^2^		47 ± 15	0.11	0.009
	Urinary protein excretion, g/24 h		0.2 (0.0–0.5)	−0.06	0.22
	Proteinuria (>0.5 g/24 h), *n* (%)		166 (27)	−0.11	0.006
Inflammation					
	hs-CRP, mg/L		2.0 (0.7–4.8)	−0.19	<0.001
	sICAM-1, ng/L		602 (514–720)	−0.17	<0.001
	sVCAM-1, ng/L		965 (772–1196)	−0.16	<0.001
Lipids					
	Total colesterol, mmol/L		5.6 ± 1.0	0.05	0.24
	HDL colesterol, mmol/L		1.0 ± 0.3	0.11	0.004
	LDL cholesterol, mmol/L		3.5 ± 0.9	0.07	0.09
	Triglycerides, mmol/L		1.9 (1.4–2.6)	−0.13	0.001
	Use of statins, *n* (%)		295 (49)	0.06	0.13
Oxidative Stress					
	Gamma glutamate, U/L		24 (18–39)	−0.10	0.02
	Alkaline phophatase, U/L		72 (57–94)	−0.21	<0.001
	Uric acid, mmol/L		0.4 (0.3–0.5)	−0.08	0.05
Glucose Homeostasis					
	Insulin, µU/mL		11 (7–16)	−0.08	0.04
	Glucose, mmol/L		4.5 (4.1–5.0)	−0.07	0.06
	HbA_1c_, %		6.5 ± 1.0	−0.12	0.002
	Diabetes, *n* (%)		105 (17)	−0.11	0.008
Hematology					
	Leukocyte count, *x* 10^9^/L		8.5 ± 2.4	−0.03	0.42
	Hemoglobin, mmol/L		8.5 ± 0.9	0.01	0.77
	Platelets count, *x* 10^9^/L		231 ± 69	−0.02	0.56

* Unadjusted. Abbreviations: ACE, angiotensin converting enzyme; CV, cardiovascular; CVA, cardiovascular accident; eGFR, estimated Glomerular Filtration Rate; HbA1c, glycated hemoglobin; HDL, high-density lipoprotein; hs-CRP, high-sensitive C reactive protein; LDL; low-density lipoprotein; m-TOR, mechanistic target of rapamycin; MI, myocardial infarction; sICAM-1, soluble intercellular cell adhesion molecule 1; sVCAM-1, soluble vascular cell adhesion molecule 1; TIA, transient ischemic attack; RTR, renal transplant recipients. Baseline characteristics normally distributed are summarized using means (SD), whereas skewed distributed variables are given as medians (IQR); percentages were used to summarize categorical variables. Multivariate linear regression analyses were performed to obtain a *p* value of potential associations of baseline characteristics of renal transplant recipients with plasma vitamin C concentration.

**Table 2:** Prospective analysis of plasma vitamin C on all-cause mortality in RTR.

	Vitamin C, Status				Vitamin C, Continuous		
	≤28 µmol/L *n* = 133			>28 µmol/L *n* = 465	2log, µmol/L *n* = 598		
	HR	95% CI	*p*	Reference	HR	95% CI	*p*
Model 1	1.95	1.35–2.81	<0.001	1.00	0.71	0.59–0.87	0.001
Model 2	1.92	1.33–2.77	0.001	1.00	0.74	0.61–0.90	0.002
Model 3	1.88	1.28–2.76	0.001	1.00	0.76	0.62–0.94	0.011
Model 4	1.91	1.30–2.82	0.001	1.00	0.76	0.62–0.94	0.012
Model 5	1.80	1.22–2.65	0.003	1.00	0.79	0.64–0.98	0.030
Model 6	1.70	1.15–2.52	0.008	1.00	0.79	0.63–0.98	0.030
Model 7	1.74	1.18–2.57	0.005	1.00	0.78	0.63–0.97	0.024

Abbreviations: RTR, renal transplant recipients; HR, hazard ratio; CI, confidence interval. Model 1: Univariate. Model 2: Age and sex adjusted. Model 3: Model 2 + adjustment for estimated Glomerular Filtration Rate, proteinuria, primary renal disease, time since transplantation, and dialysis vintage. Model 4: Model 3 + adjustment for smoking and alcohol use. Model 5: Model 3 + adjustment for diabetes mellitus. Model 6: Model 3 + adjustment for systolic blood pressure, body mass index, high density lipoprotein cholesterol, and triglycerides concentration. Model 7: Model 3 + adjustment for use of calcineurin inhibitors, use of antimetabolites, use of m-TOR inhibitors, use of induction therapy, and cumulative dose of prednisolone.

**Table 3:** Mediating effects of hs-CRP, sICAM-1, sVCAM-1 separately and combined on the association of plasma Vitamin C concentration with risk of mortality in 598 RTR according to Preacher and Hayes procedure.

Potential Mediator	Effect (Path) *	Multivariate Model **	
		Coefficient (95% CI) ^†^	Proportion Mediated
hs-CRP	Indirect effect (*ab* path)	−0.016 (−0.036; −0.004)	16% ***
	Total effect (*ab* + *c’* path)	−0.103 (−0.189; −0.010)	
sICAM-1	Indirect effect (*ab* path)	−0.018 (−0.043; −0.003)	17% ***
	Total effect (*ab* + *c’* path)	−0.103 (−0.194; −0.016)	
sVCAM-1	Indirect effect (*ab* path)	−0.015 (−0.040; −0.003)	15% ***
	Total effect (*ab* + *c*’ path)	−0.103 (−0.200; −0.015)	
Combined inflammation	Indirect effect (*ab* path)	−0.033 (−0.065; −0.012)	32% ***
	Total effect (*ab* + *c*’ path)	−0.103 (−0.191; −0.013)	

Abbreviations: hs-CRP, high sensitive C-reactive protein; sICAM-1, soluble intercellular cell adhesion molecule 1; sVCAM-1, soluble vascular cell adhesion molecule 1; RTR, renal transplant recipients; CI, confidence interval. * The coefficients of the indirect *ab* path and the total *ab* + *c’* path are standardized for the standard deviations of the potential mediators, plasma vitamin C concentration and outcomes. ** All coefficients are adjusted for age, sex, estimated Glomerular Filtration Rate, time since transplantation, primary renal disease, and proteinuria. *** The size of the significant mediated effect is calculated as the standardized indirect effect divided by the standardized total effect multiplied by 100. ^†^ 95% confidence intervals for the indirect and total effects were bias-corrected confidence intervals after running 2000 bootstrap samples.
